# Hatching of *Globodera pallida* Induced by Root Exudates Is Not Influenced by Soil Microbiota Composition

**DOI:** 10.3389/fmicb.2020.536932

**Published:** 2020-10-08

**Authors:** Camille Gautier, Lisa Martinez, Sylvain Fournet, Josselin Montarry, Jean-Claude Yvin, Eric Nguema-Ona, Anne-Yvonne Guillerm-Erckelboudt, Christophe Piriou, Juliette Linglin, Christophe Mougel, Lionel Lebreton

**Affiliations:** ^1^Institut national de recherche pour l’agriculture, l’alimentation et l’environnement (INRAE), UMR1349 IGEPP, Institute of Genetic Environment and Plant Protection, Le Rheu, France; ^2^Centre Mondial de l’Innovation-Roullier, Laboratoire de Nutrition Végétale - Pôle Stress Biotique, Saint Malo, France; ^3^Institut national de recherche pour l’agriculture, l’alimentation et l’environnement (INRAE), UMR1349 IGEPP, Institute of Genetic Environment and Plant Protection, Ploudaniel, France

**Keywords:** microbial composition, plant-parasitic nematodes, suicide hatching, potato, metabarcoding

## Abstract

Plant-parasitic nematodes are among the most harmful pests of cultivated crops causing important economic losses. The ban of chemical nematicides requires the development of alternative agroecological approaches to protect crops against nematodes. For cyst nematodes, egg hatching is stimulated by host plant root exudates. Inducing “suicide hatching” of nematode second-stage juveniles (J2), using root exudates in the absence of the host plant, may constitute an effective and innovative biocontrol method to control cyst nematodes. However, before considering the development of this approach, understanding the effect of soil biotic component on cyst nematode hatching by root exudates is a major issue. The effectiveness of this approach could be modulated by other soil organisms consuming root exudates for growth as soil microbiota, and this must be evaluated. To do that, four different native agricultural soils were selected based on their physicochemical properties and their microbiota composition were characterized by rDNA metabarcoding. To disentangle the effect of microbiota from that of soil on hatching, four recolonized artificial soils were obtained by inoculating a common sterile soil matrix with the microbiota proceeding from each agricultural soil. Each soil was then inoculated with cysts of the potato cyst nematode, *Globodera pallida*, and low or high doses of potato root exudates (PREs) were applied. After 40 days, viable J2 remaining in cysts were counted to determine the efficiency of root exudates to stimulate hatching in different soils. Results showed that (i) when physicochemical and microbiota compositions varied among native soils, the hatching rates remained very high albeit small differences were measured and no dose effect was detected and (ii) when only microbiota composition varied among recolonized soils, the hatching rates were also high at the highest dose of PREs, but a strong dose effect was highlighted. This study shows that abiotic and biotic factors may not compromise the development of methods based on suicide hatching of cyst nematodes, using root exudates, molecules inducing J2 hatch, or trap crops.

## Introduction

Crop pest management is one of the major challenges to ensure agricultural production and to cope with the expected increase of the world population. During the past decades, chemical control has been a standard and an efficient practice to control pests and prevent economic losses (Popp et al., 2013). However, the intensive use of these agrochemical products has led to the detection of chemical in the environment, loss of biodiversity in agrosystems, as well as a rise of human and animal health risks in some agricultural production areas (e.g., Carvalho, 2017). These environmental and human health concerns are at the heart of civil society debates and public health policies, leading to the withdrawal of several chemicals in many countries. It has become necessary to search for new alternative and agroecological solutions for plant protection. This is the case, in particular, for the control of plant parasitic nematodes for which many nematicides have been recently removed from the market in Europe. As a result, several crops and vegetable industry sectors are left with few solutions.

Plant-parasitic nematodes represent a worldwide concern in food security, as they often cause nonspecific symptoms resulting in lower crop yields with significant economic losses (Nicol et al., 2011; Jones et al., 2013). Root-knot (*Meloidogyne* spp.) and cyst nematodes (*Globodera* and *Heterodera* spp.) are the two most economically important and damaging of cultivated crops (Molinari, 2011). Cyst nematodes are obligatory root endoparasites, with the ability to produce a thick survival structure, the cyst, formed by the dead female body. Cysts can contain up to 200–500 juveniles that adapt to the absence of the host by remaining in dormant eggs until their host returns. Dormancy can last for several years, which thus make these pests hard to eradicate. Basically, the second-stage juveniles (J2) hatch from the cyst depending on climate conditions and perception of chemical mediators [i.e., hatching factors (HFs)] released from the host roots. The level of dependence on root exudates varies considerably between species of cyst nematodes. For instance, *Globodera pallida*, one of the most harmful pathogens of potato (*Solanum tuberosum*) and quarantine organism worldwide, is very specific to the Solanaceae family. Indeed, this species hatches only after the perception of HFs, some of which have been identified in potato root exudates (PREs), such as the solanoeclepin A (Masler and Perry, 2018; Sikder and Vestergård, 2020).

Rhizodeposits, substances released by plant roots into their surrounding environment, include water-soluble exudates (amino acids, organic acids, sugars, phenolics, and other secondary metabolites) and water-insoluble products (sloughed cells and mucilage; Badri and Vivanco, 2009). This exudation is influenced by many plant characteristics, such as plant species and plant age, and also by many other biotic and abiotic factors (Jones et al., 2004). Through the root exudates, plants mediate a number of beneficial but also deleterious interactions with soil organisms (Dakora and Phillips, 2002; Bais et al., 2006; Baetz and Martinoia, 2014). Indeed, a range of diverse soil parasites, such as bacteria, fungi, oomycetes, nematodes, protists, or insects, have synchronized their life cycle to one of their hosts to enhance their chances of infection and survival (Perry and Clarke, 1981; Chippendale, 1982; Brown and Tellier, 2011; Zwanenburg et al., 2016). Host detection mainly occurs through a broad range of signaling molecules released by roots. Some authors suggest to use this chemical signal, as biocontrol methods, to lure the parasites and thus to decrease their population in soil, as for example, for controlling parasitic weeds (Zwanenburg et al., 2016), pathogens (Rashid et al., 2013; Balendres et al., 2016), or nematodes (Devine and Jones, 2000a; Lettice and Jones, 2015). The principle of this strategy, namely suicide hatching, is to apply hatching stimulants into the soil to induce hatching of the parasite in absence of the host plant and thus its starvation and death. For instance, in potato cyst nematodes, it was evaluated that hatched J2 cannot survive more than 2 weeks in the absence of a host plant (Robinson et al., 1987). In general, biocontrol methods are very promising in laboratory studies, but they are less reliable in the field where ecological interactions, in particular with microbial communities, are much more complex than those occurring in controlled conditions (Nicot et al., 2011; Le Mire et al., 2016; Sikder and Vestergård, 2020). Some bacteria and fungi in the soil feed on carbon compounds contained in root exudates and thus could alter the perception of chemical mediators by parasites, preventing the hatching. In this way, before developing biocontrol methods based on the suicide hatching principle, using root exudates, molecules inducing J2 hatch, or trap crops, many studies are first needed in the laboratory and in the field to measure the effects of physicochemical properties and soil microorganisms on the transmission and perception of hatching chemical mediators. Devine and Jones (2001) showed that the suicide hatching of the potato cyst nematode *G. pallida* induced by the incorporation of tomato root exudates in the soil was better in sandy soils than in clay and peaty soils under field conditions without considering the effect of soil microbiota.

In this study, we evaluated the effect of soil microbiota (and physicochemical properties) on the hatching of cyst nematodes. To do so, native agricultural soils differing by their physicochemical and microbial characteristics were selected to evaluate the global soil effect. To disentangle the microbiota effect from that of soil physicochemical properties, a sterile soil matrix was used and inoculated with the microbiota proceeding from different native soils (NSs). In parallel, metabarcoding analyses were carried out to describe the bacterial and fungal compositions. Each soil was artificially inoculated with cysts of *G. pallida*, and two different doses of PREs were added by exogenous applications. At the end of the experiment, the viable J2 remaining in the cyst were counted in order to determine the efficiency of root exudates to stimulate cyst hatching according to the different soil properties.

## Materials and Methods

### Soil Materials and Inoculation

#### Native Soils

To measure the effect of soil (biological and physico-chemical effects), 10 soil types were sampled in fields from the main potato production areas in France. In each field, soil samples were collected at 10–30 cm below the surface at several sampling points distributed along a diagonal and were pooled to obtain 25 kg of soil. The soils were homogenized and subsequently stored in containers at ambient temperature in the dark. Physical and chemical properties of each soil were determined at the Capinov laboratory of agri-food, agricultural, and environmental analysis (29,800 Landerneau, France; Table 1). A principal component analysis (PCA) was performed on the properties of the 10 sampled soils (dudi.pca function, “ade4” package; Dray and Dufour, 2007; fviz_pca_var function, “factoextra” package; Kassambara and Mundt, 2017) to explore variations in the dataset according to factors known as some of the main drivers of microbial community diversity in soils. These were namely organic matter (OM), pH, and soil texture (silt, clay, and sand; Girvan et al., 2003; Lauber et al., 2008; Baker et al., 2009; Supplementary Figure S1). Geographic location was also used as a discriminant factor to avoid selecting two soil samples from the same area. Out of the 10 soils, four were retained to achieve the suicide hatching experiments. They were A (Normandy, France), D (Hauts-de-France, France), F (Brittany, France), and J (Auvergne-Rhône-Alpes, France).

**Table 1:** Physicochemical properties of the native agricultural soils. The bold lines correspond to the selected NS (A, D, F, and J) and the sterilize matrix (La Gruche), which was used for recolonization process.

Soils	pH	CO	OM	P_2_O_5_	K	Ca	Mg	Clay	Silt	Sand	Texture
		(g/kg)	(g/kg)	(g/kg)	(g/kg)	(g/kg)	(g/kg)	(g/kg)	(g/kg)	(g/kg)	
**A**	**7.4**	**16**	**27.6**	**0.19**	**0.2**	**4.97**	**0.1**	**271**	**544**	**144**	**Silty Clay**
B	8.1	14.6	25.3	0.27	0.39	11.66	0.13	297	481	187	Silty sandy clay
C	6.9	10.4	17.9	0.31	0.2	2.17	0.07	136	696	141	Silt
D	8.2	10	17.3	0.16	0.22	0.44	0.11	92	263	620	Silty Clay
E	6.2	24.8	42.9	0.47	0.28	1.46	0.14	157	527	258	Clayey sandy silt
F	6.8	20.4	35.3	0.42	0.44	3.39	0.15	157	656	141	Silt
G	7.2	20.9	36.2	0.26	0.19	2.15	0.12	124	733	94	Silt
H	6.2	6	10.4	0.42	0.12	0.73	0.08	78	110	797	Sand
I	7	10.3	17.8	0.12	0.17	2.26	0.07	160	738	77	Silt
J	6.1	19.7	34	0.17	0.06	1.3	0.06	122	167	668	Clayey Sand
La Gruche	5.75	7.34	12.67	0.04	0.20	3.71	0.63	106	470	424	Sandy silt

#### Recolonized Soils

To measure the effect of soil microbiota, the microbial communities of the four NSs A, D, F, and J were inoculated in a common sterile soil matrix. The soil used as matrix, named La Gruche, was collected at the INRAE experimental site (Pacé, 48°08′24.468” N, 01°48′0.99” W). The topsoil (0–5 cm) was removed and the layer between −5 and −30 cm was harvested, homogenized, sieved at 4 mm and mixed up with silica sand (1/3 sand and 2/3 soil). The soil was then sterilized with gamma radiation at 35 kGy (IONISOS, Pouzauges, France) to eradicate indigenous microorganisms and kept for 2 months in 5 kg plastic containers for stabilization at 18°C in the dark. The physicochemical properties, listed in Table 1, were not affected by the gamma radiation.

After 2 months, this soil was inoculated and incubated according to the method described by Lachaise et al. (2017). Briefly, the inoculation process consisted of inoculating 5 kg of the sterile soil matrix (1 container) with 90 g of NS (A, D, F, or J), re-suspending in 500 ml of deionized and twice autoclaved water. Four inoculations for each NS, corresponding to four containers, were performed per NS. Containers were incubated at 18°C in the dark to allow both bacterial and fungal growth. All containers were shaken for 5 min (Turbula®, Willy A. Bachofen) and opened for 3 min under sterile conditions (laminar flow cabinet) several days a week to facilitate microbial respiration and recolonization. Four additional containers (one of each inoculated microbiota) were added to check the biotic capacities of the recolonized soils (RSs) based on colony forming units (CFUs) count method. At the end of the recolonization process (14 days), the four containers for each NS were pooled to obtain 20 kg of RSs and pH of the four RSs were measured using the method of Sumner (1994). In brief, each soil was first dried in a stove for 48 h at 105°C. Then, 8 g of dry soil was suspended in 8 ml of CaCl2 (1:1 dilution) and agitated at 250 rpm for 30 min, and measurements were performed with a PHM220 pH meter (MeterLab®, Germany).

### Characterization of Bacterial and Fungal Communities

#### Microbial DNA Sample Preparation

Soil DNA extractions were performed from three replicate samples per NS and per RS collected at the end of the recolonization process according to the Genosol protocol (INRA UMR Agroecology, Dijon, France) as described by Plassart et al. (2012) and modified by Ourry et al. (2018). Each sample was lyophilized under a 170-h program (−40°C/40h, −20°C/60, and then −10°C/70 h) and stored at −20°C. DNA was extracted from 1 g of freeze-dried soil in 5 ml of lysis buffer containing 100 mM of Tris-HCl (pH 8), 100 mM of EDTA (pH 8), 100 mM of NaCl, 2% SDS, and sterile ultrapure water in a 15 ml Lysing Matrix E tube (MP Biomedicals, Santa Ana, CA, USA, containing 1.4 mm ceramic spheres, 0.1 mm silica spheres, and eight 4 mm glass beads). Then, tubes were shaken manually and then for 30 s at 4 m∙s^−1^ with a FastPrep® 24 (MP Biomedicals, Santa Ana, CA, USA) grinder for three times. Following vortex agitation, tubes were heated at 70°C during 30 min in a water bath (vortex agitations at 15 and 30 min) and then centrifuged at 3,500 rpm for 10 min at 20°C to prevent SDS solidification. For the next steps of DNA extraction, samples were duplicated in order to obtain a higher DNA concentration. De-proteinization was performed by adding 1/10 volume of 3 M potassium acetate (pH 5.5) to 1 ml of supernatant (two tubes per sample), and then the tubes were homogenized by turnaround and incubated 10 min on ice before being centrifuged for 10 min at 4°C and 14,000 *g*. For the precipitation step, supernatant (around 900 μl) was collected in a 2 ml tube and 900 μl (1:1) of ice-cold 100% isopropanol were added. Tubes were shaken by turnaround and kept at −20°C for 30 min. The supernatant was eliminated after centrifugation at 13,000 rpm at 4°C for 30 min, and the DNA pellets were washed with an addition of 400 μl of ice-cold 70% ethanol. Tubes were then centrifuged at 13,000 rpm at 4°C for 5 min and the supernatants were removed. The remaining traces of ethanol were eliminated in a stove at 60°C during 15–20 min. Then, the DNA pellets were suspended with 100 μl of sterile ultrapure water. The duplicated samples were pooled and stored at −20°C prior to purification. Soil samples were purified twice. The first purification required PolyvinylPolyPirrolidone (Sigma Aldrich) Microbiospin (Bio-Rad, Hercules, CA, USA) columns, which were prepared according to the protocol described by Ourry et al. (2018). For this purpose, 100 μl of DNA solution were deposited on top of a column (previously transferred to a clean tube) and centrifuged at 1,000 *g* at 10°C during 4 min after incubation on ice for 5 min. The second purification was performed using the Geneclean® Turbo kit (MP Biomedicals). For this, five volumes of Geneclean Turbo GNomic Salt Solution (GTGNSS) was added to the obtained DNA and homogenized by pipetting. The mixture was deposited on the top of a purification column from the kit, centrifuged at 10,000 *g* at 10°C during 10 s, and tubes were emptied. Then, 500 μl of Geneclean Turbo Wash (GTW) were added into the column and tubes were centrifuged (10,000 *g* at 10°C for 10 s) and emptied. This washing step was realized twice. Empty columns were centrifuged at 10,000 *g* at 10°C for 4 min. Columns were put in a new tube, 30 μl of Geneclean Turbo Elution (GTE) solution were deposited into them, and then samples were incubated on ice for 5 min before being centrifuged at 10,000 *g* at 10°C during 1 min. The GTE, incubation on ice and centrifugation steps were repeated twice to finally obtain approximately 60 μl of clean DNA. Samples were then stored at −20°C until further analysis.

DNA quantification was performed using a Quantus™ Fluorometer (Promega, Madison, WI, USA) and the Quantifluor kit (dsDNA: E26710). PCR amplifications and sequencing were performed at the GenoScreen platform (Lille, France) using the Illumina MiSeq “paired-end” 2*250 bp sequencing technology. PCR were conducted according to the sequencing platform protocol using primers pairs: Forward_479 (5′-CAGCMGCYGCNGTAANAC-3′) and Reverse_888 (5′-CCGYCAATTCMTTTRAGT-3′), and also Forward_FF390 (5′-CGATAACGAACGAGACCT-3′) and Reverse_FR1 (5′-ANCCATTCAATCGGTANT-3′) to amplify 16S and 18S rDNA genes, respectively.

#### Sequence Processing

Fastq files (read 1 and read 2) were processed with DADA2 package version 1.10.1 (Callahan et al., 2016) on the R software (R Development Core Team, 2019). The default parameters proposed by the DADA2 workflow were retained except from truncLen argument, which was set at 200. Moreover, only sequences which lengths ranging from 369 to 374 and from 312 to 324, for bacteria and fungi, respectively, were kept. Taxonomy affiliations for the amplicon sequence variants (ASVs) obtained with DADA2 were performed using a naive Bayesian classifier (Wang et al., 2007) on the Silva ver. One hundred thirty-two databases (Callahan, 2018) for bacteria and the Silva 18S ver. One hundred twenty-eight databases (Morien and Parfrey, 2018) for fungi. Unclassified phyla were removed from the dataset. Then, rarefaction curves were realized using the “ggrare” function of “ranacapa” package (Kandlikar et al., 2018) to verify that the sequence coverage was sufficient to accurately describe the bacterial and fungal composition of each sample. Data were normalized based on sequencing depth using a rarefaction procedure (rarefy_even_depth function, “phyloseq” package) at 12,614 and 14,873 per sample for bacteria and fungi, respectively. At the end of this workflow, output files obtained were an ASV table and a taxonomy table.

#### Microbial Analyses

##### Alpha Diversity

Bacterial and fungal richness and diversities, characterized as number of ASVs found in each sample and the Shannon index (on normalized data), were determined with the diversity function of R “vegan” package (Oksanen et al., 2019). For the analysis, richness and diversities were compared between NS and RS using a Wald chi-square test on a Linear Mixed-Effects Model, (lmer function, “lme4” package; Bates et al., 2015) taking into account the soil (A, D, F, and J) and the type of soil (NS and RS), as fixed factors, and the replicate, as random factor. Pairwise comparisons of estimated marginal means (EMMs) were performed (emmeans function, “emmeans” package; Lenth, 2019), applying the false discovery rate (FDR) correction for *p*-values.

##### Beta Diversity

The analyses of beta diversity were performed on normalized, filtered (using a 1‰ threshold) and log2-transformed ASVs tables. A Bray-Curtis dissimilarity matrix was calculated from ASV data using the vegdist function from the R “vegan” package. To compare the bacterial and fungal community compositions, a distance-based redundancy discriminant analysis (dbRDA) was performed on the matrix data (dbrda function, “vegan” package). Then, a type II permutation *F*-test for constrained multivariate analysis was performed on the dbRDA to evaluate the contribution of each factor (i.e., soil, type of soil, and interaction between them) to microbial community composition (“RVAideMemoire” package; Herve, 2019).

##### Taxonomic Rank Analyses

To identify phyla and genus differences among soils, normalized counts were analyzed using Likelihood Ratio Test (LR Test) on Generalized Linear Model (distribution: quasipoisson, link function: log), and pairwise comparisons of EMMs were then computed. To visualize community taxa and map similarities/differences among NS and RS, heat trees were realized with the “heat_tree_matrix” function from the R “metacoder” package (Foster et al., 2017). Heat trees were realized on normalized and filtered (genera higher than 50/10,000) data, which were transformed into presence/absence data.

### Suicide Hatching Assay

#### Biological Material

##### Nematodes

The *Globodera pallida* population “Chavornay”, multiplied in 2018 on potato cv. Désirée, was used to perform the suicide hatching experiments. Cysts were extracted from soil by a Kort elutriator and stored at 5°C in the dark before furthers experiments. Cysts were sieved between 400 and 500 μm diameters and an estimation of initial viable J2 contained per cyst (Pi) was done. To do so, and because this is a destructive method, 50 cysts were randomly selected from this pool of cysts: each cyst was crushed in 1 ml of tap water and the number of viable J2 was counted under a stereomicroscope. From this count, Pi was estimated at 290 viable J2 per cyst. Then, for the experiment, 10 cysts were put in a tulle bag that was inserted in the different treatments. The tulle mesh allowed the movement of J2 from the cyst to the soil.

##### Root Exudates

Potato root exudates were produced using 400 pre-germinated tubers of potato cv. Désirée. Tubers were suspended on grids onto 10 cans (40 tubers per can) containing 10 L of tap water each. The distance between tubers and water was approximately 0.5 cm; this close proximity of tubers to water meant that roots produced by the tubers were immediately immersed in water. Three weeks after incubation in the dark at 20°C, root exudates were collected (i.e., all the solution in the can), pooled, filtered at 0.20 μm to eliminate potential bacterial contamination and stored at −20°C. Carbon concentration of PRE, determined at the Centre Mondial de l’Innovation (CMI) Groupe-Roullier (Saint Malo, France), was 66.4 mg of C per g of dry matter. Then, as the HFs were generally C-based molecules, two doses of root exudates were tested based on the C concentration: 15 ml containing 0.13 mg of C (dose 1) and 2.5 ml (six times less) containing 0.021 mg of C (dose 2) completed with sterile permuted water at 15 ml. These two doses were selected from previous experiments performed in pots showing that applications of PRE at 30 and 15 mg of C per g of dry matter achieved a high level of hatching (unpublished data, Dr. B. Ngala and N. Mariette). So, to explore a dose effect in our experiment, we divided six times the lowest dose previously tested.

#### Hatching Assay

Suicide hatching experiments were conducted in a climatic chamber at 18/14°C day/night with 16 h photoperiod. Soils (550 g for NS and 650 g for RS) were packed in pots of 9*9*9.5 cm with a bag of 10 cysts inserted at a depth corresponding to one-third of the pot (from the bottom). Pots were disposed in randomized way into three blocks to buffer the possible inequalities of light and temperature within the chamber. Temperature and humidity of soils were measured during all the experiment, respectively, by thermic probes (External Soil Temp Sensor 3,667, Spectrum Technologies Inc.) and soil moisture sensors (WaterScout SM 100 Sensor 6,460, Spectrum Technologies Inc.) plugged into measurement station (WatchDog micro station 1,000 series, Spectrum Technologies Inc.). Temperature in climatic chamber was also measured with three thermochron buttons (iButton ds1921G, maxim integrated).

Three treatments per soil were tested: dose 1 of PRE (0.13 mg of C), dose 2 of PRE (0.021 mg of C), and negative control (sterile permuted water). Each treatment was replicated nine times (three blocks and three repetitions per block). The experiment took place over 40 days and every 4 days, pots were weighed and watered to reach 80% of their field capacity, with (i) dose 1 of PRE, dose 2 of PRE, or water as per treatment at days 0, 4, 8, 12, and 16 (five applications) and (ii) water at days 20, 24, 28, 32, and 36 (five applications).

At the end of the experiment (day 40), the bags of cyst were retrieved from each pot. Bags were opened, and cysts were crushed to count under a stereomicroscope the number of remaining (unhatched) viable J2 per cyst (Pf) as described above for Pi assessments. The hatching rate for each treatment was then computed as the ratio of difference between the mean Pi and Pf values to the mean Pi value [(Pi-Pf)/Pi] and expressed as percentages.

#### Statistical Analyses

The effects on hatching of the factors soil (A, D, F, and J), treatment (dose 1, dose 2, and control), and interaction between soil and treatment were tested using a Linear Mixed-Effects Model, (function lmer, “lme4” package; Bates et al., 2015) with parametric analysis of variance (ANOVA) provided by R package “car” (Fox et al., 2012). Block effect was introduced as random effect in the model. Normality and homogeneity of variances were checked by performing Quantile-Quantile (QQ) plots of the residuals and the fitted models using function plotresid (“RVAideMemoire” package; Herve, 2019). Pairwise comparisons of EMMs were performed (“emmeans” package; Lenth, 2019), applying the false discovery rate (FDR) correction for values of *p*.

## Results

### Diversity and Structure of Microbial Communities

The suicide hatching experiments were conducted on four NSs and four RSs obtained by inoculating the same soil matrix with the microbiota from the NSs. Two factors have been considered: (i) the type of soil, namely NS and RS, and (ii) the soil, namely A, D, F, and J corresponding to the global properties (biotic and abiotic) for NSs and to the microbiota for RSs.

After normalization of metabarcoding data by rarefaction, 12,614 and 14,873 reads per sample were obtained for bacteria and fungi, respectively. Alpha diversity of native and recolonized soils was assessed by evaluating the number of ASV and calculating the Shannon index (Supplementary Figure S2). In bacterial communities, the number of ASVs was influenced by the type (NS and RS) of soil (*χ*^2^ = 335.56, df = 1, *p* < 0.0001), the soil (*χ*^2^ = 23.48, df = 3, *p* < 0.0001), and the interaction between type of soil and soil (*χ*^2^ = 21.96, df = 3, *p* < 0.0001). Indeed, there were significantly more ASVs in NS (mean of 950 ± 37.21) than in RS (mean of 493 ± 19.10; Supplementary Figure S2A). Moreover, among NS, soil F contained the highest number of ASVs (1,122 ± 13.33) and soil A the lowest (836 ± 42.40). The number of ASVs among RSs was not significantly different. The Shannon index of bacterial communities was also influenced by the type of soil (*χ*^2^ = 1960.43, df = 1, *p* < 0.0001), the soil (χ^2^ = 82.44, df = 3, *p* < 0.0001) and the interaction between type of soil and soil (*χ*^2^ = 33.72, df = 3, *p* < 0.0001) with the NS (6.38 ± 0.05) containing significantly more diverse bacterial communities than RS (5.11 ± 0.05; Supplementary Figure S2B). Among NS, F was the most diverse, followed by D, J, and A, the least diverse one. In fungal communities, the number of ASVs was influenced by the type of soil (*χ*^2^ = 15.41, df = 1, *p* < 0.0001), the soil (χ^2^ = 27.55, df = 3, *p* < 0.0001), and the interaction between type of soil and soil (*χ*^2^ = 20.21, df = 3, *p* < 0.0001). Among the eight soils, the J NS contained significantly more ASVs (193 ± 7.42) than the others (Supplementary Figure S2C). Furthermore, among NS, J contained significantly more ASVs (193 ± 7.42) than D and F (132 ± 4.18 and 140 ± 1.76, respectively). For RS, no significant difference was observed. The Shannon index of fungal communities was impacted by the type of soil (*χ*^2^ = 31.19, df = 1, *p* < 0.0001) and the interaction between type of soil and soil (*χ*^2^ = 23.04, df = 3, *p* < 0.0001). Among NS, no significant difference was observed, whereas among RS, J was significantly less diverse (1.86 ± 0.22) than F (3.39 ± 0.04; Supplementary Figure S2D).

**Figure 1:**
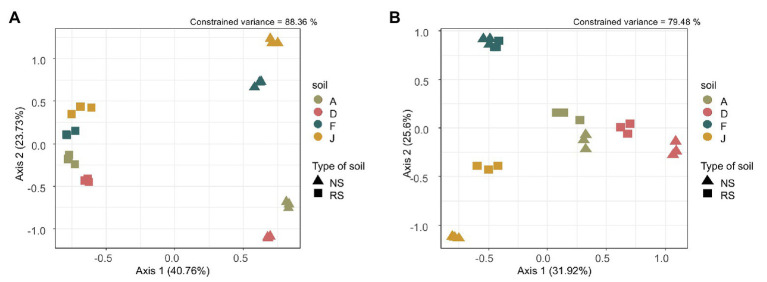
**(A)** Bacterial and **(B)** fungal community composition represented by an ordination plot and analyzed using a distance-based redundancy discriminant analysis (dbRDA) performed on the Bray Curtis distance index.

Beta diversity, i.e., reflecting bacterial community composition between soils, was driven by the type of soil (*F* = 49.18, df = 1, *p* = 0.001), the soil (*F* = 15.10, df = 3, *p* = 0.001) and the interaction between type of soil and soil (*F* = 9.00, df = 3, *p* = 0.001). Our model explained 88.36% of the total constrained variance, with 40.76% supported by axis 1 and 23.76% by axis 2, which could be explained by the type of soil (NSs vs. RSs) and the soil, respectively (Figure 1A). The bacterial community composition was different among NSs with F close to J and A to D, whereas for RSs, all bacterial communities have converged toward a more similar composition. The fungal community composition was driven by the type of soil (*F* = 13.72, df = 1, *p* = 0.001), the soil (*F* = 13.83, df = 3, *p* = 0.001), and the interaction between type of soil and soil (*F* = 2.25, df = 3, *p* = 0.004). Our model explained 79.48% of the total constrained variance, with 31.92% supported by axis 1 and 25.60% by axis 2, explained by the type of soil (NS vs. RS) and the soil, respectively (Figure 1B). Composition of fungal communities among NS varied, and differences were also observed among RS (unlike for bacterial communities). In both cases, for bacterial and fungal communities, the three replicates were grouped, indicating homogeneity in sample profiles.

### Taxonomic Composition of Microbial Communities

**Figure 2:**
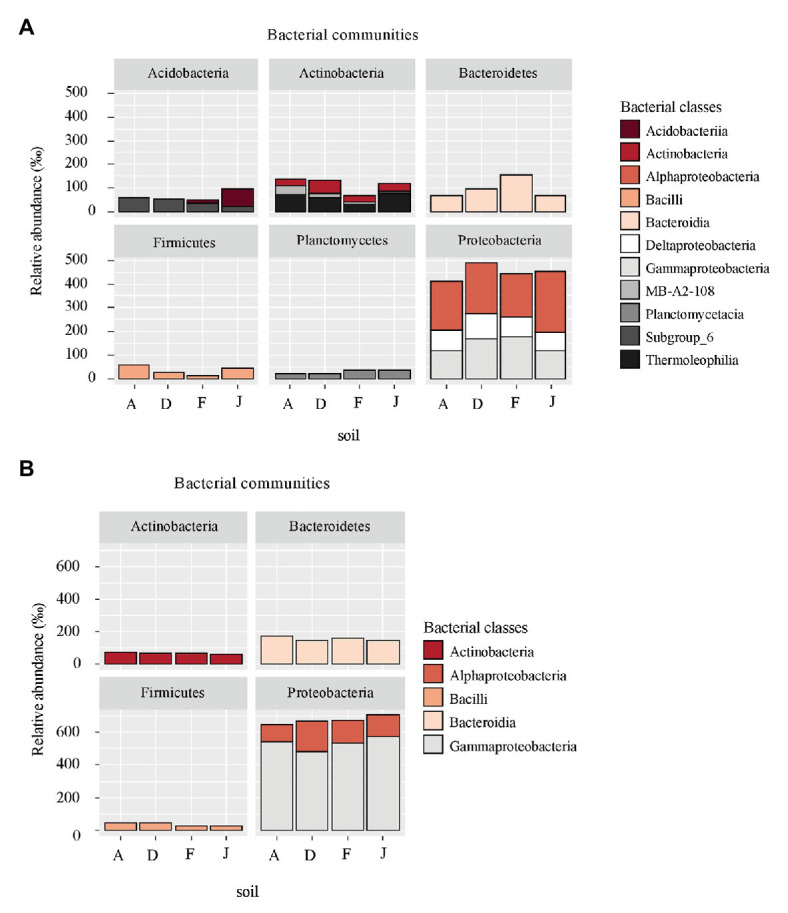
Principal bacterial classes (higher than 60‰ relative abundance) and associated phyla for **(A)** native soil (NS) and **(B)** recolonized soil (RS).

**Figure 3:**
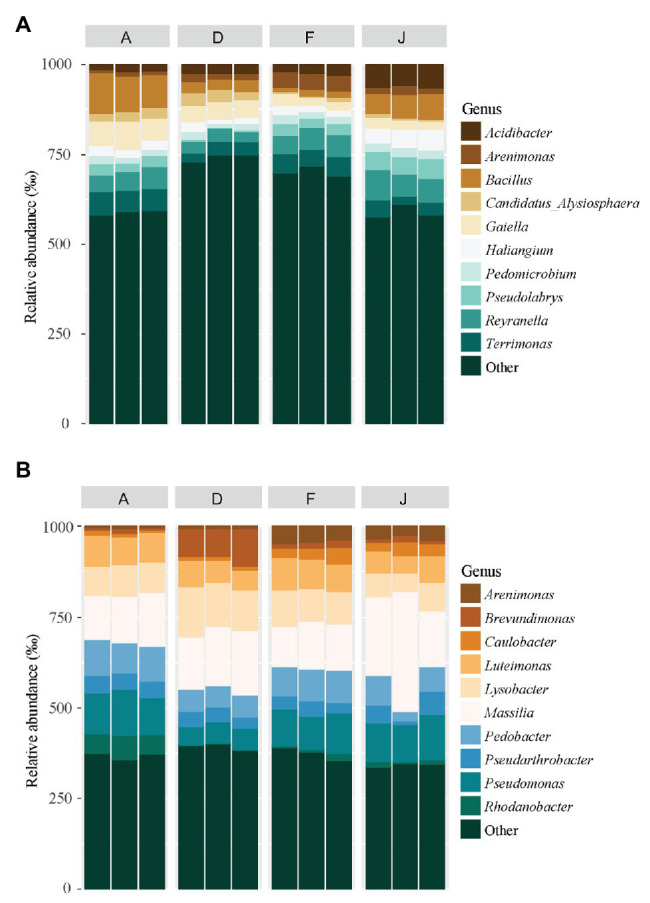
Principal bacterial genera for **(A)** NS and **(B)** RS. Unclassified genera were removed from the plot.

According to the taxonomic composition, bacterial communities were composed of 7,552 ASVs considering all NSs and RSs. The three main phyla were Proteobacteria (2,814 ASVs, 57.6% of reads), Bacteroidetes (1,214 ASVs, 13.3% of reads), and Actinobacteria (795 ASVs, 9.6% of reads). α-Proteobacteria, Bacteroidia, and Termoleophilia were the most abundant classes in NS (Figure 2A) and γ-Proteobacteria, Bacteroidia, and Actinobacteria in RS (Figure 2B). A total of 428 bacterial genera were detected. For NS, the most abundant genera were *Reyranella* (α-Proteobacteria), *Bacillus* (Firmicute), *Terrimonas* (Bacteroidetes), *Gaiella* (Actinobacteria), and *Acidibacter* (γ-Proteobacteria; Figure 3A). All soils were significantly different in terms of abundance for these genera (Supplementary Table S1A). Moreover, only J soil contained genus *Rhodomicrobium* (α-Proteobacteria; Supplementary Figure S3A). Recolonized soils had a different bacterial composition than NSs. Indeed, the most abundant genera were *Massilia* (β-Proteobacteria), *Pseudomonas* (γ-Proteobacteria), *Pedobacter* (Bacteroidetes), *Lysobacter* (γ-Proteobacteria), and *Pseudarthrobacter* (Actinobacteria; Figure 3B). Only relative abundances of *Massilia*, *Pseudomonas*, and *Lysobacter* were significantly different among the four RSs (Supplementary Table S1B). Also, only RS J contained genus *Collimonas* (γ-Proteobacteria; Supplementary Figure S3B).

**Figure 4:**
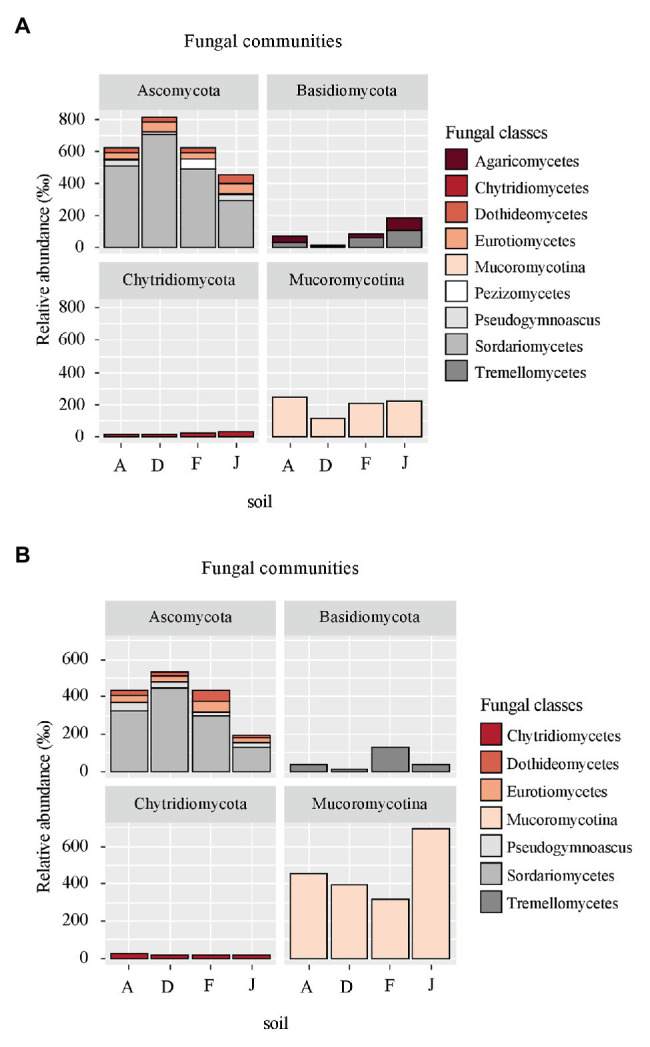
Principal fungal classes (higher than 60‰ relative abundance) and associated phyla for **(A)** NS and **(B)** RS.

**Figure 5:**
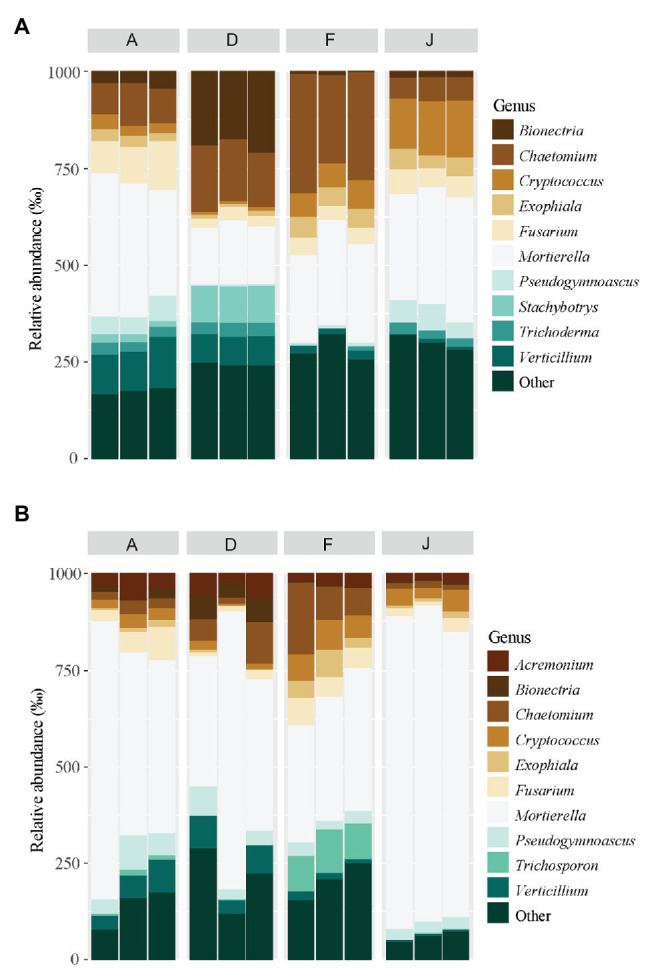
Principal fungal genera for **(A)** NS and **(B)** RS. Unclassified genera were removed from the plot.

Fungal communities were composed of 960 ASVs, considering all native and recolonized soils. The three main phyla were Ascomycota (391 ASVs, 54.5% of reads), Basidiomycota (180 ASVs, 8.8% of reads), and Mucoromycotina (80 ASVs, 33.1% of reads). Sordariomycetes, Tremellomycetes, and Mucoromycotina were the most abundant classes (Figure 4) for both NS and RS. Considering all soils, a total of 208 fungal genera were detected. In NSs, the most common genera were *Mortierella* (Mucoromycotina), *Chaetomium* (Ascomycota), *Bionectria* (Ascomycota), *Cryptococcus* (Basidiomycota), and *Fusarium* (Ascomycota; Figure 5A). All these genera were significantly different in terms of relative abundance among NSs (Supplementary Table S2A). Moreover, some genera were observed only in one soil (Supplementary Figure S4A), namely *Ambispora* (Glomeromycota) for soil A; *Stachybotrys* (Ascomycota), *Torula* (Ascomycota), and *Cochliobolus* (Ascomycota) for soil D; *Ustilago* (Basidiomycota) and *Scopulariopsis* (Ascomycota) for soil F; and *Metarhizium* (Ascomycota), *Atractospora* (Ascomycota), *Rhodocybella* (Basidiomycota), and *Dictyostelium* (Basidiomycota) *Mariannaea* (Ascomycota) for soil J. The fungal genus compositions of RS were nearly similar to NS. Indeed, *Mortierella* (Mucoromycotina), *Chaetomium* (Ascomycota), *Pseudogymnoascus* (Ascomycota), *Cryptococcus* (Basidiomycota), and *Fusarium* (Ascomycota) were the most common genera in both soil types (Figure 5B). All these genera were significantly different in terms of abundance among RSs except for *Pseudogymnoascus* (Supplementary Table S2B). Moreover, private genera were *Stachybotrys* (Ascomycota), *Torula* (Ascomycota) for RS D and *Otidea* (Ascomycota), *Ustilago* (Basidiomycota), and *Dendryphion* (Ascomycota) for RS F (Supplementary Figure S4B).

Alpha and beta diversity, with taxonomic composition, indicated a different microbiota composition among the four NS and RS.

### Effect of Soils on Suicide Hatching of Cyst Nematodes

**Figure 6:**
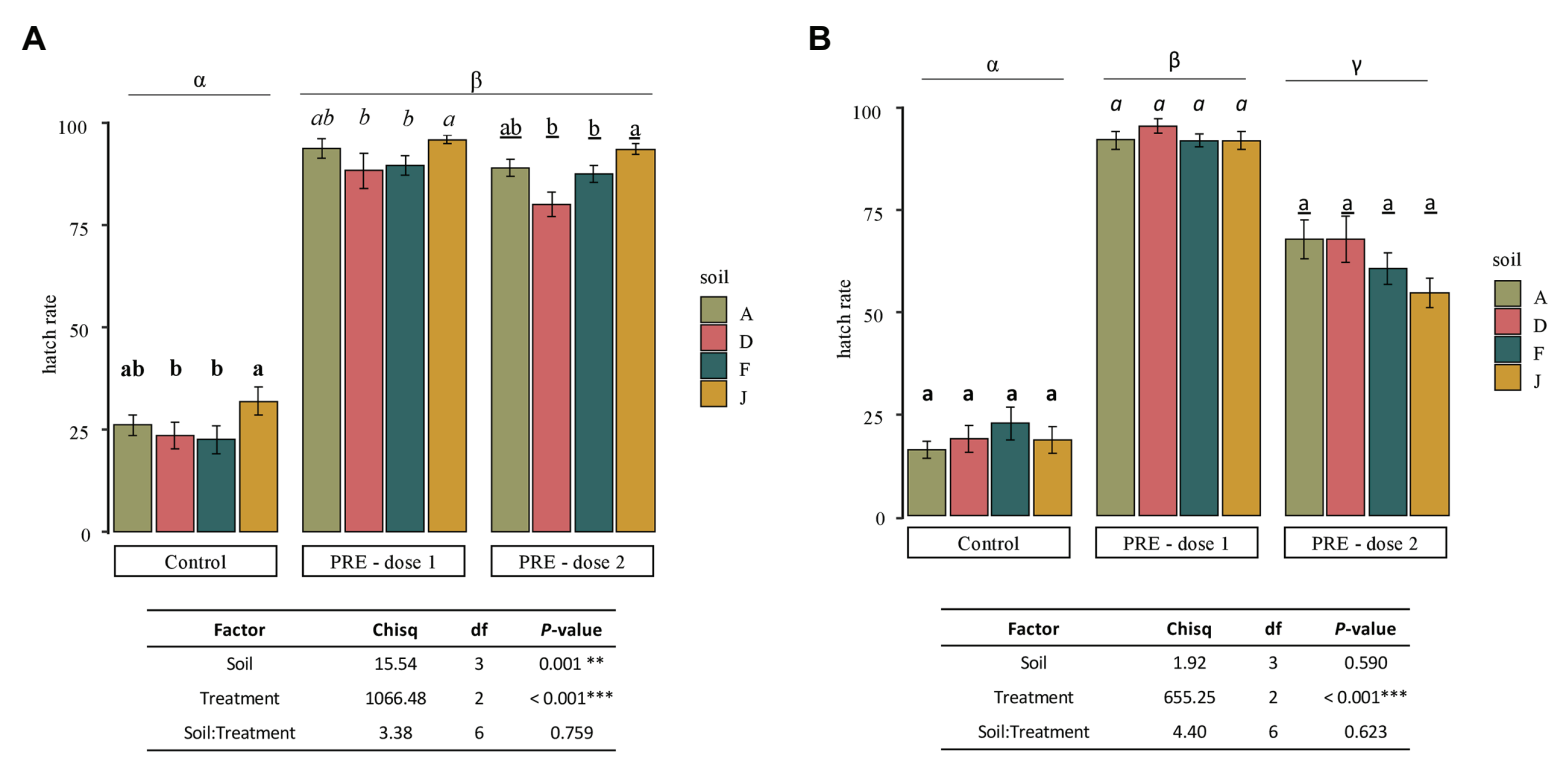
Mean of hatching rate of *Globodera pallida* juveniles (mean values ± SEM) at the end of the experiment (after 40 days) for control (water application), dose 1 (PRE application at 0.13 mg of C), and dose 2 (PRE application at 0.021 mg of C) treatments, in **(A)** NS and **(B)** RS. Letters represent significant differences between soil for a given treatment and the Greek letters among treatments.

After 40 days inside each NS or RS and application of PREs at two doses (0.13 and 0.021 mg of C), all bags of 10 cysts were retrieved from the soils and hatching rates were determined. Results clearly showed that application of PREs significantly stimulated *G. pallida* hatching, whatever the type of soil (Figure 6).

For NSs (Figure 6A), in the control (i.e., no application of root exudates), a mean of 25.95% ± 3.08 of J2 hatched spontaneously (hatch in water). For any given soil, application of PREs, dose 1 (0.13 mg of C per application) or dose 2 (0.021 mg of C per application) induced statistically the same level of hatching rate among NSs with slight but significant differences. Results indicated that, despite microbiota and physicochemical properties, which were different among NSs, the suicide hatching of the cysts was only slightly affected by the global properties of soils with no impact of the concentration of root exudates tested.

For soils that were inoculated (RS; Figure 6B), the hatching rates were not significantly different among controls (18.68% ± 3.14), dose 1 (91.37% ± 3.09), or dose 2 (61.63% ± 3.05). Nevertheless, dose 1 or dose 2 of PREs induced statistically higher hatching of nematodes than in control, and higher hatching rates were observed with dose 1 than dose 2. Thus, as RSs were different in bacterial and fungal community composition, the microbiota does not affect the hatching of cysts, in the experimental conditions tested.

## Discussion

The main goal of this study was to evaluate the impact of NSs (physicochemical properties and microbiota) and soil microbiota on the hatching of *G. pallida* induced by PREs. Our results showed that the global properties of soils and the microbiota did not affect, in our conditions, the efficiency of root exudates to induce hatching of *G. pallida*, in the absence of host plants.

The first step of this study was to select four contrasting native agricultural soils in terms of soil texture, physicochemical properties, and microbiota. Physicochemical properties of soils were used as discriminant factors to select soils, as previous studies have revealed that soil texture, organic C content, and pH are the main drivers of microbial communities (Fierer and Jackson, 2006; Lauber et al., 2008; Naveed et al., 2016). For instance, regarding soil characteristics to explain the distribution of soil bacteria richness in France, pH is positively correlated to richness, but clay content, C:N ratio, and total K negatively correlated, according to the French Soil Quality Monitoring Network (Terrat et al., 2017). Moreover, other parameters such as climate conditions or land use can influence microbial distribution (Dequiedt et al., 2011; Maestre et al., 2015). Thus, the geographical location was also considered. Consistently with this hypothesis, the rDNA metabarcoding analysis confirmed differences in microbiota composition among the NSs in terms of diversity, abundance, and bacterial and fungal communities.

The second step was to obtain soils with the same physicochemical properties and different microbiota. For this, a common sterile soil matrix was inoculated with microbiota from the four selected NSs. All the four RSs shared, hence, the same physicochemical properties. The process of recolonization slightly affected the physicochemical properties of the matrix as exemplified for pH, which initially was 5.75 for the sterilized matrix and ranged between 5.83 and 6.07 after recolonization. However, both bacterial and fungal communities were significantly, but contrastingly, affected by habitat changes. The major modification occurred for bacteria communities with a decrease of specific richness and species diversity in recolonized compared to NSs. Moreover, the relative abundances of the same bacterial genera, such as *Massilia* (β-Proteobacteria), *Pseudomonas*, and *Lysobacter* (γ-Proteobacteria) increased during the recolonization process and as a consequence, a convergence of bacterial composition from all RSs was observed. These genera are excellent competitor microorganisms and can colonize rapidly an uncrowded environment (Bowen and Rovira, 1976; Ofek et al., 2012). Conversely, the fungal communities were less impacted in terms of alpha diversity and conserved a similar composition as in NSs but some slight differences, i.e., an increase of *Mortierella* (Mucoromicota) relative abundances, were observed. Regarding the whole microorganism communities (bacteria and fungi), four different microbiota were obtained during the recolonization process.

Our main goal was to anticipate potential fluctuations of hatching rate according to both physicochemical properties and microbiota or microbiota only. The hatching process of cyst nematodes is a complex chain reaction, which depends, according to the considered nematodes species on several different stimuli and environmental conditions, such as temperature, pH, soil moisture, and texture. These abiotic parameters are not the only factors, which influence the hatching but also the host plant cultivar, plant age, or the period of exudates production (Evans, 1983; Perry and Gaur, 1996). In our assay, some of these factors were controlled to favor the hatching. Indeed, temperature and moisture were maintained in a range close to the optimum described in the literature (see Masler and Perry, 2018; for a review): 14–18°C for temperatures and 80% of field capacity for moisture, and PRE was produced during 3 weeks after potato germination.

In NS, results showed a very high level of suicide hatching of *G. pallida*, compared to the control (water applications) when PRE was applied regardless of tested dose and soil. Five applications of dose 1 (0.13 mg C) or 2 (0.021 mg C), every 4 days, induced the same level of hatching. Hatching factors are present in very small amounts in root exudates. For example, Fukuzawa et al. (1985) found 1.25 mg of glycinoeclepin A as its bis(p-bromophenacyl) ester (HF for *Heterodera glycines*, the soybean cyst nematode) in 1058 kg (dry weight) of bean roots. Also, Devine and Jones (2000b) found a HF in PRE at less than 2.9 × 10^−5^% of recovered OM, active *in vitro* at less than 2.1 × 10^−8^ M. Some authors reported a dose-response relationship between PRE and the stimulation of hatching (Devine et al., 1996; Fukuzawa, 2007). Due to the high specificity of *G. pallida* to PRE, it seems to be interesting to test even smaller doses to highlight an effect and determine the sensitive threshold. However, slight but significant differences were observed among soils. Indeed, for dose 1, dose 2, or control, soil J had a minor but significantly higher hatching than soils D and F. Some authors highlighted the role of microorganisms on nematode hatching in soils and suggested a tritrophic interaction between host, microorganisms, and cyst nematodes (Ryan et al., 2003; Ryan and Jones, 2003). Ryan and Jones (2003) demonstrated that the spontaneous hatching of potato cyst nematodes (in the absence of plant or PRE) was higher in sand compared to *in vitro*, suggesting the occurrence of other HF produced by microorganisms. Lettice and Jones (2015) showed that isolated rhizobacteria, including *Bacillus sp.*, induced higher levels of *G. pallida* hatching in the absence of host plant than control. As soils A and J contained higher levels of *Bacillus*, we cannot exclude the hypothesis that soil microorganisms play a role in hatching and may be implicated, for an undetermined proportion, in the spontaneous hatch of cyst nematodes.

In the recolonized matrix, our results showed a high hatching compared to the control with no effect of microbiota origin but a non-negligible impact of tested doses. Indeed, dose 2, characterized by smaller quantity of C than dose 1, induced a lower hatching of *G. pallida*. Root exudates HF include C-based molecules, such as solanoeclepin A (C27H30O9), glycinoeclepin A (C25H34O7), α-solanine (C45H73NO15), or α-chaconine (C45H73NO14), which constitute an important source of food for microorganisms living in soil (Bertin et al., 2003; Bais et al., 2006; Mendes et al., 2013). Moreover, microorganisms can be characterized according to their life-history strategy: copiotroph (r-strategist) or oligotroph (K-strategist). According to Fierer et al. (2007), copiotroph microorganisms have high growth rates in response to availability of abundant C resources and are able to colonize rapidly an unexploited environment. In contrast, oligotrophs have slow growth rate and are good competitors in low nutrient environment due to their high affinity for resources. Our experimental strategy, i.e., colonization of a germ-free environment with higher nutrient resources than in NSs, created a favorable habitat to select copiotroph microorganisms. Many members of the β- and γ-Proteobacteria, Bacteroidetes, and Actinobacteria are classified as copiotrophs (Fierer et al., 2007, 2012) and they were the main phyla in RSs. The increase of copriotroph bacteria in RS compared to NS, with more equilibrates ecological attributes (copiotroph-oligotroph continuum), could explain the dose effect observed. The C from PRE present in soils was probably largely metabolized by the selected bacterial r-strategists, leaving few hatching signals for the nematodes. Another hypothesis is the physicochemical properties or the structure of the soil matrix that we used, a sandy silt soil, could influence the availability of HFs in the soils. For this, it would be interesting to test another structure of soil matrix.

To conclude, our study provides key elements for the development of suicide hatching, as a promising eco-friendly control strategy, against *G. pallida* in NSs. Even if our RSs are artificial soils, they indicate that applications of a small C amounts can decrease the HF efficiency in presence of many copiotroph microorganisms. The selection of copiotroph microorganisms could occur in relation to soil management (tilling and organic amendment) or during different stresses which reduced microbial biomass or diversity (Ho et al., 2017; Chen et al., 2018). For furthers studies, it would be interesting to evaluate the impact of root exudates on microbial communities in relation to nematode hatching rate.

## Data Availability Statement

The raw metabarcoding data sets are available on the European Nucleotide Archive database system under the project accession number PRJEB36768. Soil sample accession numbers range from ERS4307830 to ERS4307877 and sequencing runs from ERR3908564 to ERR3908611. An Excel file containing the raw data of the suicide hatching, in native and recolonized soils, is available at data.inrae.fr (doi: 10.15454/THNCAC).

## Author Contributions

CG, LM, SF, A-YG-E, CP, JL and LL performed the experiments according to a protocol elaborated jointly by CG, SF, JM, J-CY, EN-O, CM, and LL. CG, LM, SF, JM, and LL analyzed the data. CG, SF, JM, CM and LL wrote the text and prepared the figures. All authors contributed to the article and approved the submitted version.

### Conflict of Interest

The authors declare that the research was conducted in the absence of any commercial or financial relationships that could be construed as a potential conflict of interest.
