# Supplementary material for: Hatching of Globodera pallida Induced by Root Exudates Is Not Influenced by Soil Microbiota Composition
Source: Front Microbiol. 2020 Oct 8;11:536932. doi: 10.3389/fmicb.2020.536932 (PMC7578397; doi:10.3389/fmicb.2020.536932)
Supplement: Supplementary file 1 [file Data_Sheet_1.pdf]

Supporting Information – Figure S1: PCA on the physicochemical properties of the native agricultural soils. Colours (brown, red, blue and orange) indicate the selected NS (A, D, F and J).

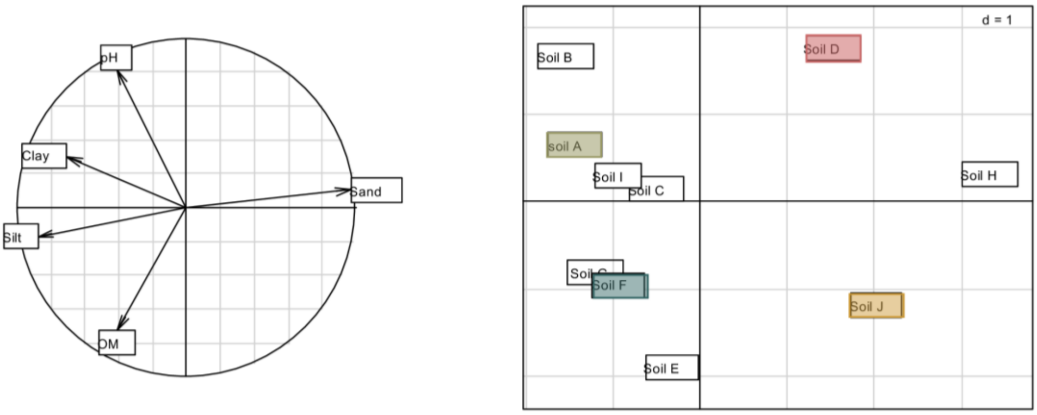

Supporting Information – Figure S2: Alpha diversity in NS and RS represented by the (**A**) bacterial / (**C**) fungal specific richness (i.e. observed ASV) and the diversity of (**B**) bacterial / (**D**) fungal species (i.e. Shannon index). Letters represent significant differences between soils for a given type of soil.

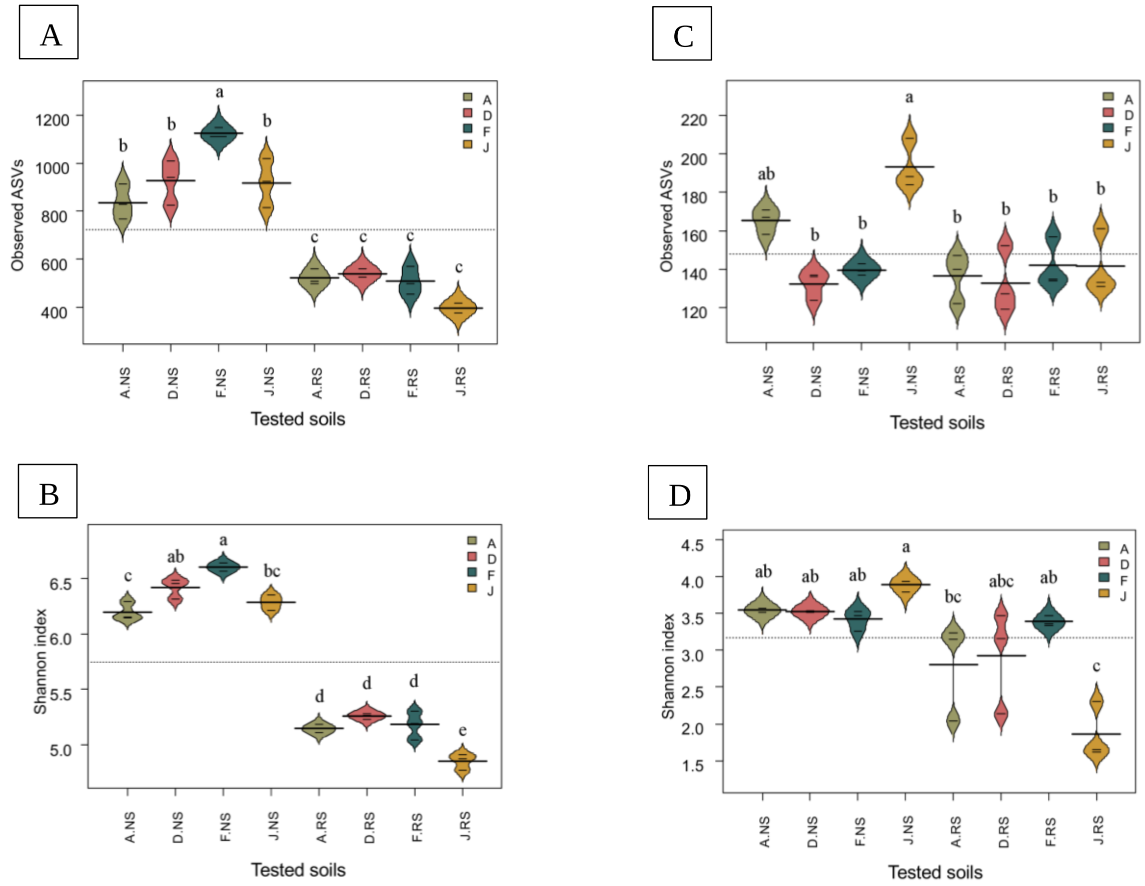

Supporting Information – Table S1: Statistical output of the ten most abundant bacterial genera. Numbers below each soil specify the statistical groups obtained from pairwise comparisons. Bold p-values indicate a significant difference (**A**) among NS and (**B**) among RS.

A

**Genus**	P_value	Soil A	Soil D	Soil F	Soil J
Acidibacter	**0.000**	1	2	2	3
Arenimonas	**0.000**	1	2	3	2
Bacillus	**0.000**	4	2	1	3
Candidatus_Alysiosphaera	**0.000**	2	3	1	1
Gaiella	**0.000**	3	2	1	1
Haliangium	**0.000**	1	1	1	2
Pedomicrobium	0.089	1	1	1	1
Pseudolabrys	**0.000**	2	1	2	3
Reyranella	**0.000**	2	1	23	3
Terrimonas	**0.000**	2	1	2	1

B

**Genus**	P_value	Soil A	Soil D	Soil F	Soil J
Arenimonas	**0.00**	1	2	4	3
Brevundimonas	**0.00**	1	2	1	1
Caulobacter	**0.00**	1	1	2	2
Luteimonas	**0.03**	1	1	1	1
Lysobacter	**0.00**	2	3	2	1
Massilia	**0.01**	1	12	1	2
Pedobacter	0.07	1	1	1	1
Pseudarthrobacter	0.82	1	1	1	1
Pseudomonas	**0.00**	2	1	2	2
Rhodanobacter	**0.00**	3	1	2	2

Supporting Information – Table S2: Statistical output of the ten most abundant fungal genera. Numbers below each soil specify the statistical groups obtained from pairwise comparisons. Bold p-values indicate a significant difference (**A**) among NS and (**B**) among RS.

A

**Genus**	P_value	Soil A	Soil D	Soil F	Soil J
Bionectria	**0.000**	3	4	1	2
Chaetomium	**0.000**	2	3	4	1
Cryptococcus	**0.000**	2	1	3	4
Exophiala	**0.000**	2	1	3	3
Fusarium	**0.000**	3	1	1	2
Mortierella	**0.000**	3	1	2	3
Pseudogymnoascus	**0.000**	2	1	1	2
Stachybotrys	**0.000**	2	3	1	123
Trichoderma	**0.000**	2	2	1	2
Verticillium	**0.000**	4	3	2	1

B

**Genus**	P_value	Soil A	Soil D	Soil F	Soil J
Acremonium	**0.022**	2	2	12	1
Bionectria	**0.000**	2	3	1	1
Chaetomium	**0.003**	1	12	2	1
Cryptococcus	**0.000**	2	1	3	23
Fusarium	**0.006**	2	1	2	1
Mortierella	**0.003**	12	12	1	2
Pseudogymnoascus	0.052	1	1	1	1
Stachybotrys	**0.000**	1	2	1	12
Trichosporon	**0.000**	2	1	3	1
Verticillium	**0.000**	2	2	1	1

Supporting Information – Figure S3: Heat trees comparing bacterial taxa (genera which the abundance is higher than 50/10000) (**A**) between NS and (**B**) between RS. A coloured taxon (blue or red) indicates the presence of this taxon (at least in two replicates on three) into a given soil. Purple taxa are present (at least in two replicates on three) in the two compared soils. Unclassified genera were removed from the plot.

A

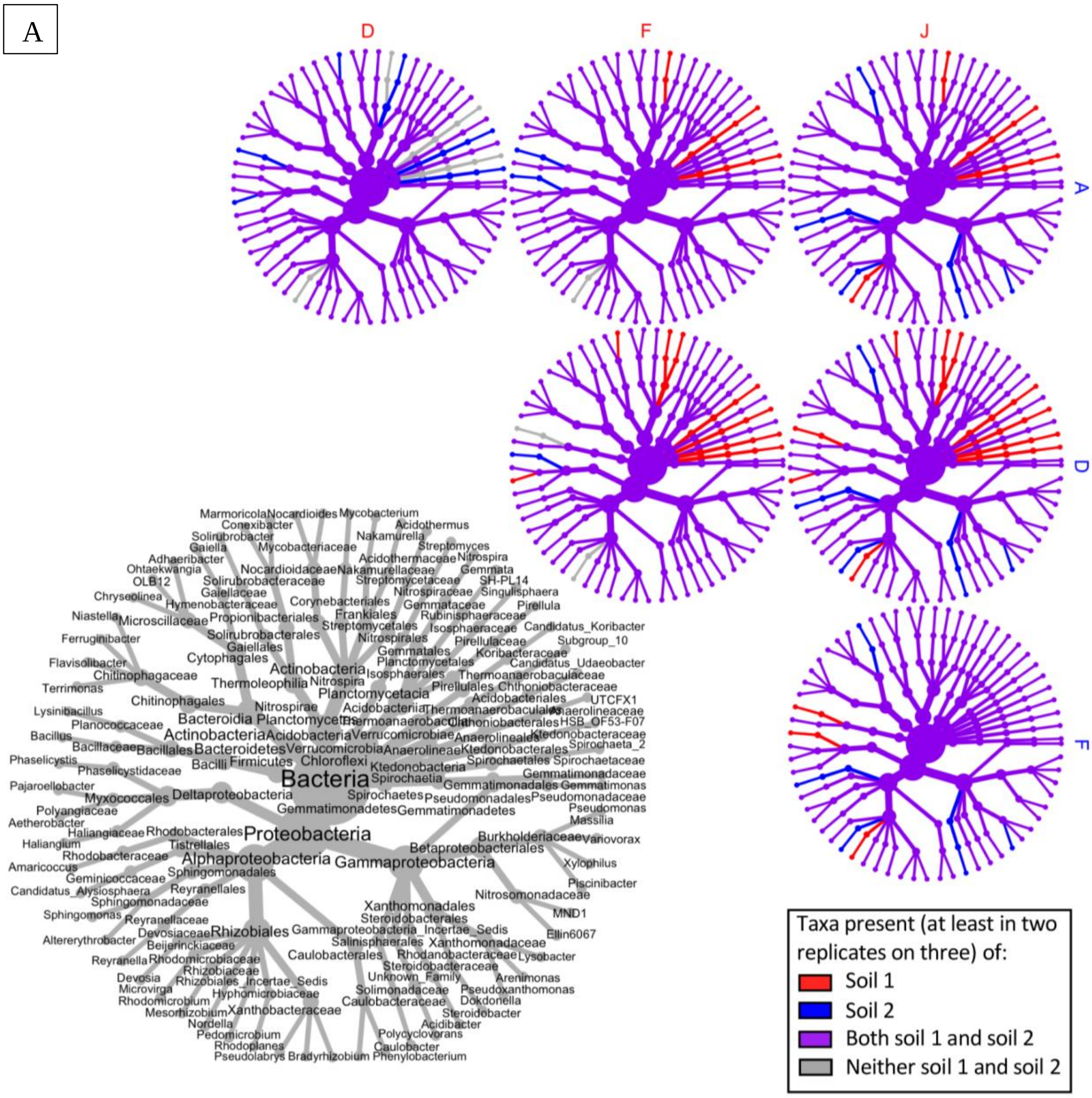

B

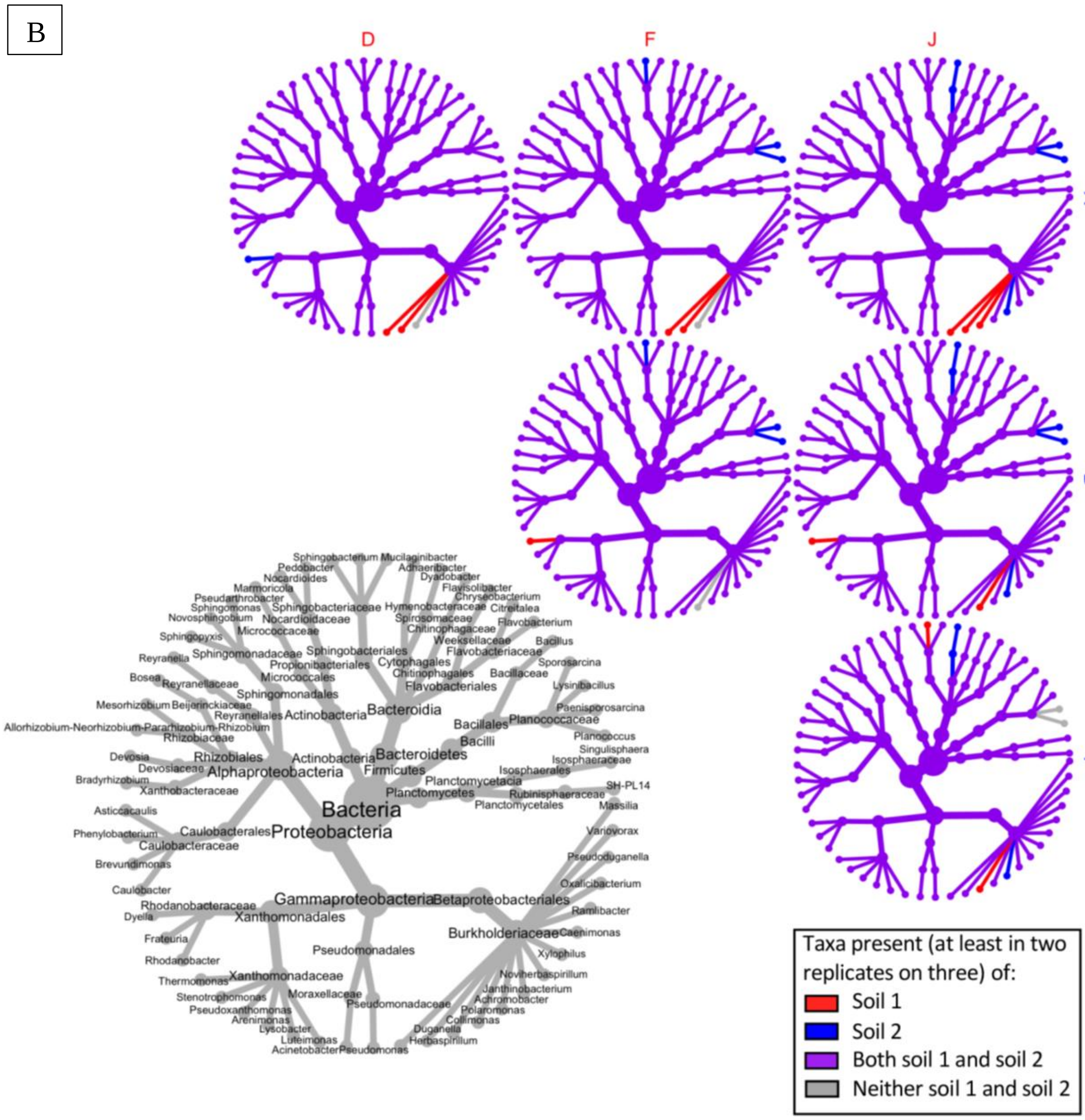

Note: Soil 1 is used for soils with red letters (i.e. D, F and J) and soil 2 is used for soils with blue letters (i.e. A, D, F).

Supporting Information – Figure S4: Heat trees comparing fungal taxa (genera which the abundance is higher than 50/10000) (**A**) between NS and (**B**) between RS. A coloured taxon (blue or red) indicates the presence of this taxon (at least in two replicates on three) into a given soil. Purple taxa are present (at least in two replicates on three) in the two compared soils. Unclassified genera were removed from the plot.

A

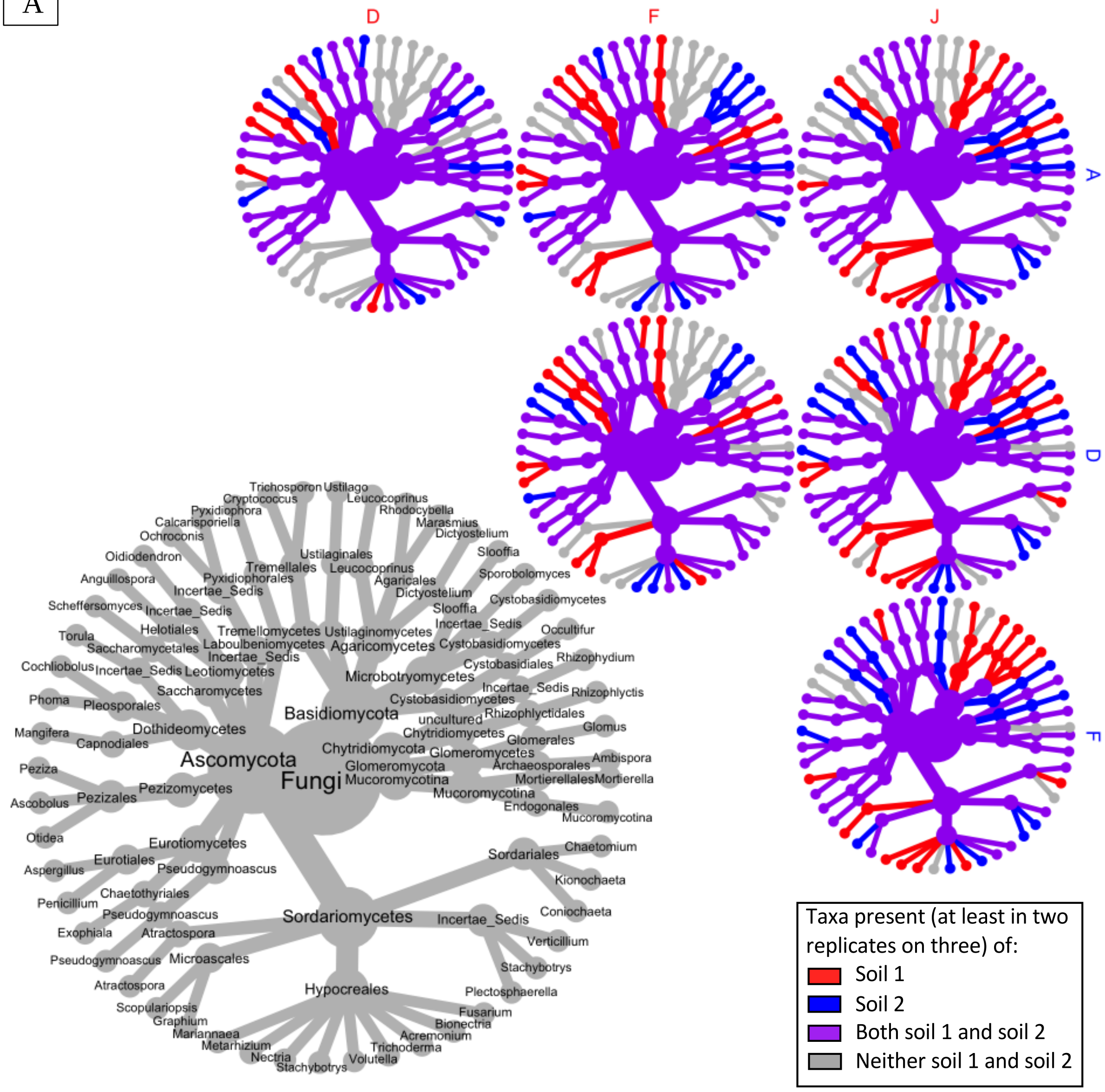

B

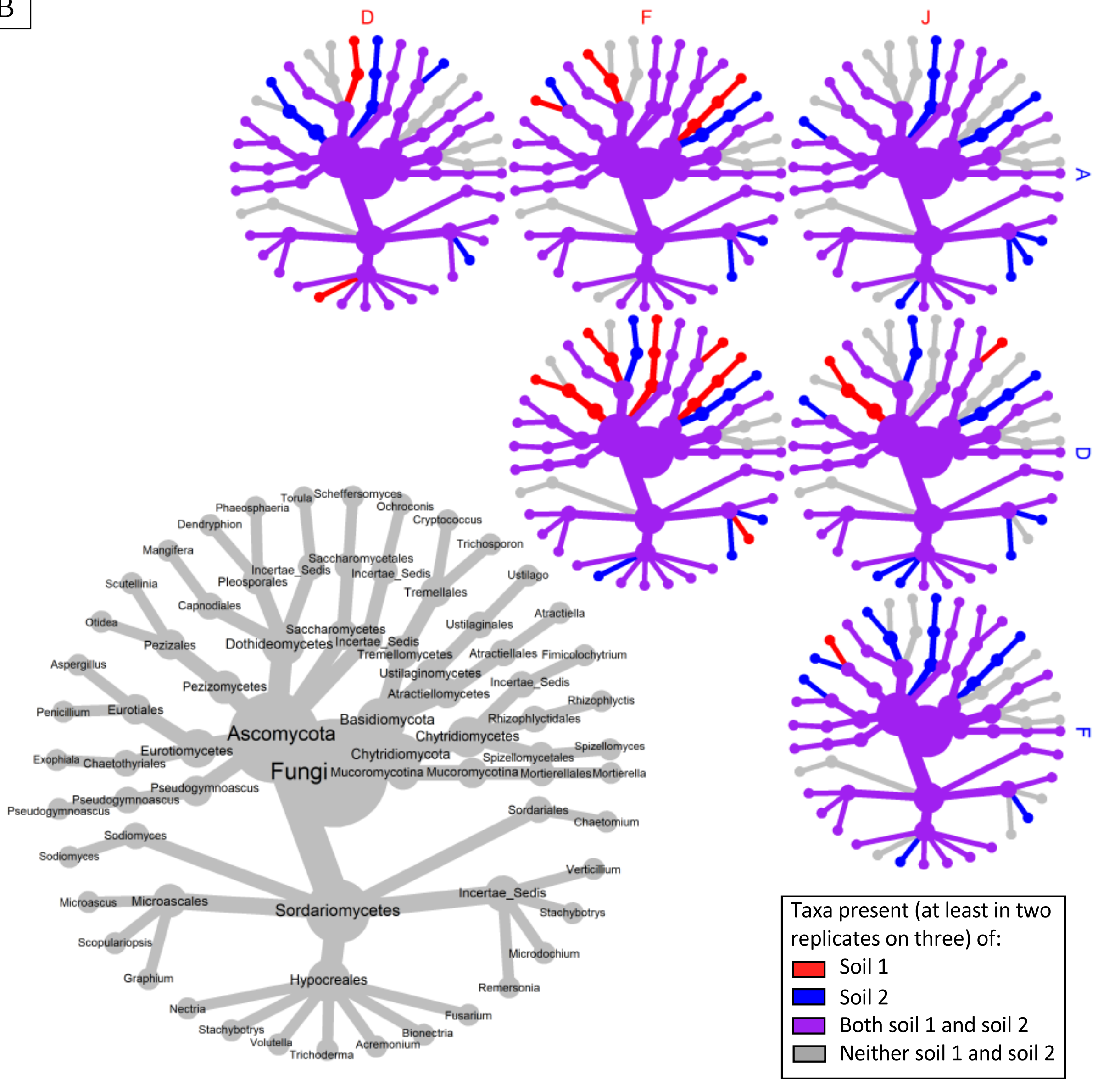

Note: Soil 1 is used for soils with red letters (i.e. D, F and J) and soil 2 is used for soils with blue letters (i.e. A, D, F).
